# Molecular Dynamics Simulations of the Human Glucose Transporter GLUT1

**DOI:** 10.1371/journal.pone.0125361

**Published:** 2015-04-28

**Authors:** Min-Sun Park

**Affiliations:** Janelia Research Campus, Howard Hughes Medical Institute, Ashburn, Virginia, United States of America; Hong Kong University of Science and Technology, HONG KONG

## Abstract

Glucose transporters (GLUTs) provide a pathway for glucose transport across membranes. Human GLUTs are implicated in devastating diseases such as heart disease, hyper- and hypo-glycemia, type 2 diabetes and caner. The human GLUT1 has been recently crystalized in the inward-facing open conformation. However, there is no other structural information for other conformations. The X-ray structures of *E*. *coli* Xylose permease (XylE), a glucose transporter homolog, are available in multiple conformations with and without the substrates D-xylose and D-glucose. XylE has high sequence homology to human GLUT1 and key residues in the sugar-binding pocket are conserved. Here we construct a homology model for human GLUT1 based on the available XylE crystal structure in the partially occluded outward-facing conformation. A long unbiased all atom molecular dynamics simulation starting from the model can capture a new fully opened outward-facing conformation. Our investigation of molecular interactions at the interface between the transmembrane (TM) domains and the intracellular helices (ICH) domain in the outward- and inward-facing conformation supports that the ICH domain likely stabilizes the outward-facing conformation in GLUT1. Furthermore, inducing a conformational transition, our simulations manifest a global asymmetric rocker switch motion and detailed molecular interactions between the substrate and residues through the water-filled selective pore along a pathway from the extracellular to the intracellular side. The results presented here are consistent with previously published biochemical, mutagenesis and functional studies. Together, this study shed light on the structure and functional relationships of GLUT1 in multiple conformational states.

## Introduction

Glucose is the main source of energy for all eukaryotic cells. It is transported across the membrane by Glucose Transporters (GLUTs), which are membrane proteins belonging to the Sugar Porter family of the Major Facilitator Superfamily (MFS). The MFS is one of the largest membrane protein families whose members are present across all kingdoms of life [1, 2]. In humans GLUTs are expressed in all cell types where they are primarily responsible for sugar uptake destined for metabolic pathways [3, 4]. GLUTs are facilitators therefore glucose is transported down its concentration gradient across the membrane without coupling to an ion. Among the various GLUT isoforms, glucose transporter 1 (GLUT1) is most widely expressed and is present in blood cells and detected throughout the brain [5]. Glucose transporter 1 deficiency syndrome results from impaired glucose transport across the blood-brain barrier [6–8]. Patients often suffer from seizures and display delayed development, acquired microcephaly, hypotonia, ataxia, and dystonia [8]. Studies have identified several GLUT1 mutations contributing to this disease with phenotypes ranging from mild to severe perturbations in GLUT1 function: N34 [9]; S66 and T310 [10]; G91 [11]; R126 [12]; R153, E247 and K256 [13]; E146 and R333 [14]; T295 [15]; P383 and R400 [16]. Moreover, most cancers over express GLUT1 for enhanced glucose metabolism to support uncontrolled cellular proliferation [17]. Recently, the human GLUT1 have been crystallized in the inward-facing conformation (open to the cytoplasm) with a presence of *n*-nonyl-β-D-glucopyranoside (β-NG) molecule at 3.2 Å resolution [18].

The proton coupled xylose permease (XylE) from *Escherichia coli* transports D-xylose against its concentration gradient from the periplasm to the cytoplasm [19, 20]. XylE shows sequence identity of about 30% and a sequence similarity of about 60% with human GLUT1 making it one of the closest bacterial homologues to the human glucose transporter. As with other members of the MFS, XylE consists of 12 transmembrane (TM) helices where helices 1–6 form the N-terminal domain while helices 7–12 form the C-terminal domain. The sugar-binding site is located at the interface between these two domains. It is understood that substrate translocation takes place as the N- and C-terminal domains rock against one another, transitioning XylE from the outward-facing (open to the periplasm) to the inward-facing (open to the cytoplasm) conformation to deliver the substrate from one side of the membrane to the other [21, 22]. This rocker switch mechanism [21, 22] appears to be common to all members of the MFS.

XylE is the only MFS member that has been crystallized in multiple conformational states that include both the partially occluded outward-facing and the inward-facing open conformations [23, 24]. Moreover, the outward-facing conformation includes D-xylose or D-glucose at the substrate-binding site [23]. The residues important for binding D-glucose in the crystal structure of XylE are all conserved in the human GLUT1 with the exception of Gln415, which is Asn411 in GLUT1, even though XylE does not transport D-glucose. XylE can be superimposed with the human GLUT1 in a RMSD (root-mean-squared deviation) of ~1.9 Å over aligned Cα atoms in the inward-facing conformation (the flexible cytoplasmic domains were not included). Given the high structure and sequence identity between XylE and GLUT1, and the fact that multiple high-resolution structures have been determined for XylE in different conformations, we reasoned that a reliable homology model for GLUT1 in the outward-facing conformation could be constructed based on XylE and the substrate translocation in GLUT1 during the conformational transition can be analyzed by molecular dynamics simulations.

Here, we constructed a homology model for human GLUT1 in the partially occluded outward-facing conformation based on the available structure of XylE. Following equilibration, all-atom molecular dynamic simulations enabled the GLUT1 model to further open toward the extracellular side, which allowed us to identify a new fully opened outward-facing conformation. Comparing the new conformation to the X-ray structure in the inward-facing open conformation, we addressed the role of the flexible intracellular domains. Moreover, we visualized and dissected for the sequence of events leading to glucose transport starting from sugar binding to sugar release in GLUT1. The simulation data was well correlated with available biochemical, mutational and functional data in the literature to enhance our understanding of GLUT1 structure-function relationships.

## Methods

### Simulation System Setup

We built homology-based models of the human glucose transporter 1 (GLUT1) using MODELLER[25, 26]. The crystal structure of XylE in the partially occluded outward-facing conformation (PDB: 4GBZ) was used as a template for the model. Sequence alignment between XylE and GLUT1 was performed using CLUSTALW[27]. Four amino acids at N-terminus were omitted in our model because this part is missing in the crystal structure of XylE. Also the extra domain at the C-terminus in GLUT1 (24 amino acids from Gln469 to Val492) was treated as a free loop due to the lack of template because it is a missing part in available crystal structures. The C-terminal loop region was not included in restraints for further MD simulations. The C-terminal tail of the GLUT1 is not critical for glucose transport. When the 24 amino acids were deleted, the level of glucose transport activity was unaffected [28]. To validate the outward-facing conformation model, a GLUT1 model in the opened inward-facing conformation was also constructed using the crystal structure (PDB: 4QIQ) of XylE as a template with the same modeling method, and compared to the experimental structure of GLUT1 (PDB: 4PYP) in the same conformation. All missing parts in the crystal structure of XylE (eight amino acids at N-terminus, Ser229 to Asn246, His262 to Gly268, and the 32 amino acids at C-terminus) were treated as free loops. A Ramachandran plot was generated and analyzed by RAMPAGE [29].

Protonation states of the titratable residues were assigned based on the pKa values calculated with the H++ server (http://biophysics.cs.vt.edu/H++), a computational prediction system for the pKa values of ionizable groups in macromolecules based on the Poisson-Boltzman (PB) or generalized Born (GB) models [30]. The protonation states of each residue were assigned based on the pKa calculations at pH 7. The model protein was incorporated perpendicularly into a 100 Å X 100 Å POPC bilayer in the xy-plane. Overlapping lipids were removed and TIP3P water molecules [31] were filed along the z-axis with 40 Na^+^ and 54 Cl^-^ ions in a 100 Å x 100 Å x 136 Å box while keeping the net charge of the system neutral. The RESP charges of POPC [32, 33] and D-glucose were derived using RED version 3 [34].

### Conventional Molecular Dynamics Simulation without Sugar

With initial configurations without sugar, the waters, ions, and lipids in the box were initially minimized by 5000 steps of steepest descent minimization followed by another 2500 steps of conjugated gradient minimization, keeping the protein constrained to their initial position. Thereafter, the entire system was minimized without restraints by the same protocol. In the next step, the temperature of the system was gradually increased with Langevin dynamics [35] from 0 to 300 K at constant volume, keeping the lipid and protein restrained with a force constant of 10 kcal‧mol^-1^‧Å^-2^. A short 2ns relaxation simulation was performed with imposing restraint energy potential of 20 kcal‧mol^-1^‧Å^-2^ for all of the peptide atoms. Then, a 67ns equilibrium simulation was performed without restraints. The last snapshot was used as an initial structure of the further steered molecular dynamics (SMD) simulation for sugar binding. To investigate the conformational change of GLUT1 without bound sugar, an additional 140ns simulation was performed, resulting in a total 207ns conventional MD simulation. To test reproducibility, all simulations were repeated with random seeds. Unless otherwise stated, all simulations here used the same protocol: All bonds involving hydrogen atoms were constrained using the SHAKE algorithm. MD simulations were carried out using the AMBER package (version 12) employing ff99SB force field combined with GAFF force field [35, 36] at constant temperature (300 K), constant pressure (1 atm) with anisotropic pressure coupling, and periodic boundary conditions. The electrostatic interactions were calculated using the particle mesh Ewald (PME) method [37] with a nonbonded cutoff distance of 10 Å. A time step of 2 fs was used and a snapshot of the system for every 5 ps was analyzed.

### Accelerated Molecular Dynamics Simulation without Sugar

The last snapshots obtained from the previous two trials of 207ns conventional MDs were used as the input structures for the Accelerated molecular dynamics (AMD) [38] simulations. In this application, dihedral angle (γ) rotations are accelerated by adding “boost potential”, Δ*V* = (*E*−*V*(*γ*))^2^ / (*α* + *E*−*V*(γ)) where *V*(γ) is the original dihedral potential, *E* is the threshold dihedral energy specified by the user and α is the acceleration parameter for the shape of the modified potentials [39]. A set of acceleration parameters, *E* and α, was used in this simulations: 16,213 and 340 kcal‧mol^-1^. These parameters were calculated from the previous conventional MD. Each AMD simulation was performed for 100ns.

### Steered Molecular Dynamics Simulations for Sugar Binding

A steered molecular dynamics (SMD) [40] simulation was performed using the last trajectory of the partially occluded outward-facing conformation obtained from the previous equilibrium MD (at 67ns). A D-glucose molecule was initially added (the C4 of the glucose was facing the extracellular face) at about 25 Å toward the extracellular side along the z-axis from Trp388, a sugar-binding residue that is located at the bottom of the substrate-binding pocket in the crystal structure of XylE (PDB: 4GBZ), and conflicted water molecules with sugar were removed. The system was minimized twice by 5000 steps of steepest descent minimization followed by another 2500 steps of conjugated gradient minimization with/without constraining the protein. The glucose was then gradually pulled down into the pore. 10ns SMD was performed introducing a 1.07 kcal‧mol^-1^‧Å^-2^ external harmonic potential to the distances (3.0 Å of the distance restraint) of five hydrogen bonds between the glucose and the four sugar-binding residues in GLUT1 in order to induce ligand fit. All these bonds were described in the XylE crystal structure (PDB: 4GBZ). The hydrogen bonds are: (1) O1 atom of glucose and NE1 atom of Trp388. (2) O1 atom of glucose and NE2 atom of Gln282. (3) O3 atom of glucose and ND2 atom of Asn288. (4) O4 atom of glucose and OD1 atom of Asn288. (5) O6 atom of glucose and NE2 atom of Gln161. In the final step, a conventional MD (CMD) without restraints was performed for 190ns.

### Targeted Molecular Dynamics Simulations for Conformational transition

A Targeted molecular dynamic (TMD) [41] simulation was employed to accelerate the conformational transition of GLUT1 in the sugar transport cycle. The form of the steering force is *V* = (*k* / 2*N*)[*RMSD*(*t*)−*RMSD*_*o*_(*t*)]^2^, where *k* is the force constant, *N* is the number of atoms, *RMSD*(*t*) is the RMSD of the simulation structure at time t relative to the prescribed target structure, and *RMSD*_*o*_ (*t*) is the prescribed target RMSD value at time t. The TMDs began from the partially occluded outward-facing conformations with glucose bound, which was obtained from the previous 10ns SMD followed by the equilibrium CMD (at 20ns). Following a 5000 step steepest descent minimization, the external force was applied on all the Cα atoms of residues 9–455 to force them into the target structure, which was the original X-ray crystal structure in the opened inward-facing conformation. A force constant of 2.0 kcal‧mol^-1^‧Å^-2^ per Cα atom was used for the initial model to gradually decrease the value of RMSD from ~10.0 Å to 0 Å during the 10ns TMD simulation. We used the same simulation parameters as used in previous conventional MD but all TMD simulations were performed using the NAMD [42], the Nosé**–**Hoover thermostat [43] was used for pressure control (piston period of 0.2 ps and piston decay time of 0.05 ps), and for non-bond interactions, the switching function began at 10 Å and the cut-off was set to 12 Å. A snapshot of the system for every 1 ps was analyzed. The last snapshot was used for the further SMD simulations for sugar release. To test if the data is reliable and reproducible, three trials with random seeds were performed.

### Steered Molecular Dynamics Simulations for Substrate Release

It began from the last trajectory from the 10ns TMD simulation for the conformational transition. Ten steered molecular dynamics (SMD) simulations were performed to release the glucose substrate with random seeds. The distance between the C2 of glucose molecule and CD of Leu260, which is at the intracellular linker between the N- and C-domain of protein (the pulling direction was determined by the vector between two appointed atoms), was gradually decreased during the simulations from ~25 Å to ~16.5 Å (pulling distance was ~ 8.5 Å). The constant pulling rate of about 0.7 Å‧ns^-1^ was applied. To prevent drifting of GLUT1 during the pulling process, the Cα atoms of residues 24 and 306, which locate far away from the binding pocket, and the backbone atoms of residue Leu260 were restrained with the 500 kcal‧mol^-1^‧Å^-2^ harmonic potential. The Potential of Mean Force (PMF) was estimated based on the Jarzynski’s equality ⟨*exp*[−*W* / *k*_*B*_ *T*]⟩ = *exp*[−Δ*G* / *k*_*B*_ *T*], where *W* is the total nonreversible work done on the system, *ΔG* is the free energy change between the initial and final states, T is temperature, and *k*_*B*_ is the Boltzman constant [44, 45]. In addition, the linear extrapolation and the cumulative integral extrapolation method [46] were used for constructing the PMF from SMDs. 500 times of calculation for the extrapolation were performed to obtain the average and standard deviation of PMF. A per-residue decomposition of the sugar binding free energy was performed using MMPBSA (Molecular Mechanics-Poisson Boltzmann Surface Area) to evaluate a contribution from each residue during the sugar release. MMPBSA computes the binding free energy by a thermodynamic cycle as a sum of the gas-phase energies, solvent free energies and entropic contributions averaged over snapshots of the MD simulation. Non-polar solvation energy terms were calculated using a generalized Born (GB) model [47] [48]. More technical details for MMPBSA computing and per-residue free energy decomposition were well described in the previous publication[49].

## Results and Discussion

### GLUT1 model construction and validation

**Fig 1:**
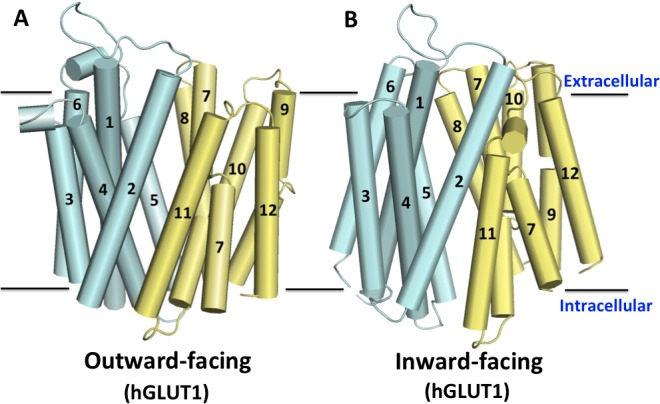
Homology-based models of human GLUT1 in (A) the outward-facing and (B) the inward-facing conformation. The flexible cytoplasmic domains are omitted. The each 12 trans-membrane (TM) domain is labeled. TM1-6 form the N-domain (cyan) and TM7-12 form the C-domain (yellow).

Two initial models of human GLUT1 were constructed based on the available XylE crystal structures (PDB: 4GBZ and 4QIQ) with the same protocol. The first XylE template was in the partially occluded outward-facing state at 2.8 Å resolution and the second was in the fully opened inward-facing conformation at 3.5 Å resolution (Fig 1A and 1B).

In general, homology modeling can correctly predict the target structure when the target structure is closely homologues to the template that was used. To evaluate our GLUT1 model in the partially occluded outward-facing conformation, the inward-facing conformational model was compared to the known X-ray crystal structure of the human GLUT1 in the same conformation (PDB: 4PYP). The inward-facing conformational model showed a close agreement with the X-ray structure. They were superimposed within the Cα RMSD value of 1.9 Å (the cytoplasmic domains were excluded). Overall, the transmembrane (TM) domains were very similar but some variations were found in loop regions (S1A Fig). The residues composing the substrate binding site in the model were also well aligned to those in the X-ray structure (S1B Fig). An analysis of Ramachandran plot showed that only three amino acids (L57, K117, and S265) in loop regions have a disallowed geometry (data not shown). We assumed that the partially occluded outward-facing model might be more reliable because the template, the X-ray structure of XylE, is in higher resolution. In addition, the thermally equilibrated structure by following MD simulations would be more favorable.

### Conventional and accelerated molecular dynamics simulations for the outward-facing model

When the conventional equilibrium MD simulation was performed on the GLUT1 partially occluded outward-facing conformational model without substrate, interestingly, the RMSD value compared to the starting model over all Cα atoms kept slightly increasing (S2A Fig). We reasoned that the partially occluded outward-facing model would favor conformational changes into a fully opened outward-facing conformation without sugar bound. We then extended the simulation an additional 140 ns conventional MD simulation (total 207 ns). In addition, to enhance the sampling, additional accelerated MD (AMD) simulation was performed for 100ns in an effort to reach for the fully equilibrated GLUT1 conformation. Indeed, the GLUT1 morphed into a fully opened conformation (S2B Fig). This MD results suggest that the fully opened outward-facing conformation may be more energetically favorable in GLUT1 in its apo state.

**Fig 2:**
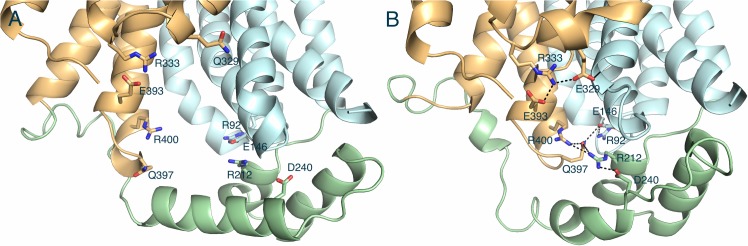
Comparison of interactions between TM domains and intracellular helices (ICH) domain of GLUT1. (A) the opened inward-facing (X-ray structure). (B) the opened outward-facing conformation (the result of the CMD simulation). The N-terminal TM domain, C-terminal TM domain, and ICH domain are shown in cyan, orange, and green, respectively. The possible hydrogen bonds and the corresponding residues are described as dashed black lines and sticks, respectively.

The intracellular linker between TM6 and TM7 folds into 3 α-helices (referred to as helices IC1-IC3). Together these soluble intercellular helices (ICH) form a bundle with another short helix from the C-terminus (helix IC4). GLUT1 and XylE appear to share similar conformation of the ICH bundle, even though the IC4 was invisible in the GLUT1 X-ray crystal structure [18]. This bundle shows numerous interactions with TM domains in the partially occluded outward-facing conformation of XylE but none of these interactions were found in the inward-facing open conformation of GLUT1. Thereby its functional role has been implied to stabilize the outward-facing conformation[18]. However, one conformation of GLUT1 offers only limited clues about this notion. We investigated the interactions at the interface between the TM domains and ICH domain in the GLUT1 model in the fully opened outward-facing conformation obtained from the CMD simulation. Indeed, significant interactions and molecular details of the interaction network were observed in the interface (Fig 2B).

Most notably, two hydrogen bond sets appeared to form the inter-domain contacts between N- and C-terminal domain, one links residues E393, R333, E329 and the backbone of the residue G138 at TM 4 domain, and the other links residues R400, Q397, E146, and R92. The residue R212 at the ICH seems to form three hydrogen bonds with D240 (at ICH domain), R400 and Q397 (at C-terminal domain), further stabilizing the second set of hydrogen bonds. This data supports the notion that the ICH domain likely stabilizes the outward-facing conformation. Also they are all in excellent agreement with the previous mutational and functional studies. Exchanging of the corresponding residues that facilitate the interaction network remarkably reduced GLUT1 transport activities: R92L, E146Q(D), E329Q, E393Q, R333L, and R400L [50]; R92W [51]; E146K [52]; R212C(H) [53]; E146K and R333W[14]; R400C[16]. Specially, it was suggested that the point mutation of E329, E393, and R400 could arrest the GLUT1 in an inward-facing conformation [50]. In fact, mutant E329Q was locked and crystalized in the inward-facing conformation [18] (Fig 2A). All genetic mutations on the residues 92, 146, 212, 333, and 400 cause glucose transporter type 1 deficiency syndrome [29, 51, 53, 54]. Therefore, our MD data strongly suggest that these mutations may weaken the set of interactions among the N-terminal, C-terminal, and ICH domain and disturb the substrate-induced conformational changes during the glucose transport.

### Steered molecular dynamics (SMD) simulation for sugar binding

XylE was crystallized with xylose or glucose [23]. All of the residues that directly interact with the glucose in XylE are conserved in human GLUT1. These residues in GLUT1 are Gln161, Gln282, Asn288, Trp388 and Asn 411 (the last is Gln415 in XylE and is the only non conserved substrate-binding residue). Most of sugar-binding residues originate from the C-terminal domain with the exception of Gln161. Independent studies showed that mutation of these residues in GLUT1 severely diminished glucose uptake indicating that they are crucial for function [55–58].

Steered molecular dynamics (SMD) [40] simulations were used to induce glucose binding and translocation into the substrate binding site from the extracellular side of the partially occluded outward-facing GLUT1 model, which was equilibrated for 67 ns by a conventional MD (CMD) simulation. The external harmonic potential was applied during the 10ns SMD to form a total of 5 hydrogen bonds between the glucose and the residues shown in the X-ray structure of XylE (Asn411 in GLUT1 was not included in this calculation). Then, an additional 190 ns CMD simulation without restraints, during which time the bound sugar was stable and maintained the interactions with the binding residues in the binding pocket, was performed (S3A and S3B Fig). This CMD simulation in the sugar binding system did not provide a large conformational change as shown in the apo system during the simulation time, which implies that sugar binding holds the GLUT1 into the partially occluded outward-facing conformation (S3B Fig) and enhances the conformational change slowly toward the inward-facing conformation (S3A and S4B Figs).

**Fig 3:**
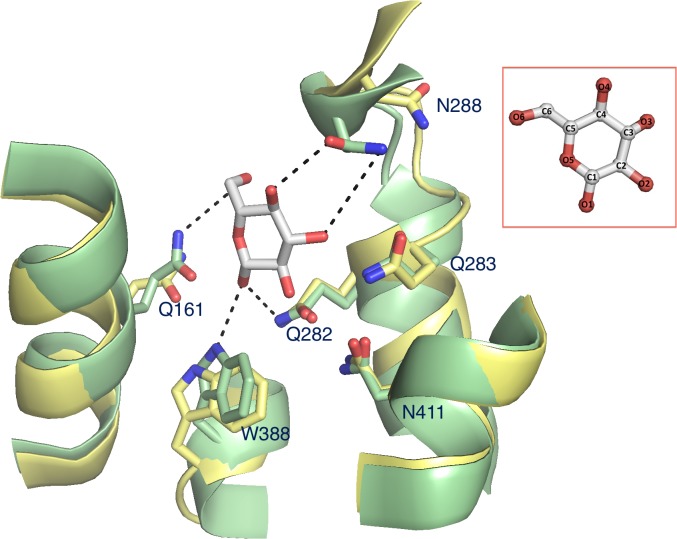
A substrate induced fit of the glucose-binding site from the fully opened outward- to the partially occluded outward-facing conformation. The initial and final locations of each binding residue are shown in yellow and green, respectively. Putative hydrogen bonds, which were used for restraints in the SMD simulation, are shown as dash lines.

The last snapshot was compared to the fully opened outward-facing conformation obtained from the previous long CMD simulation without sugar to investigate the induced substrate fit in the sugar-binding pocket. In this comparison, large rearrangements of the substrate-binding residues were observed upon sugar binding (Fig 3).

Residues underneath the sugar, Q161, W388 and Q282 on the intracellular half of GLUT1 rearrange slightly, but N288 from above the sugar to the extracellular side appears to swing ~3 Å away and flip by about 120**°** in its χ_2_ angle toward the glucose molecule. This suggests that once the sugar enters the substrate-binding site the transporter rearranges itself to close the extracellular vestibule.

**Fig 4:**
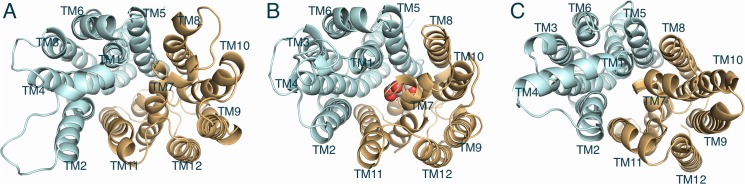
A sequence of closing or opening of extracellular vestibule. (A) The fully opened outward-facing conformation (the result of CMD and AMD simulations). (B) The partially occluded outward-facing conformation (the result of SMD and CMD for sugar binding). (C) The X-ray structure of GLUT1 in the opened inward-facing conformation. For clarity, the intracellular domains between TM6 and TM7, and the C-terminal loop domain are omitted. Bound glucose molecule is described by white (carbon) and red (oxygen) spheres. The N-domain and C-domain are shown in cyan and orange, respectively.

Compared to the X-ray structure in the inward-facing open conformation, the trajectories in the partially occluded outward-facing conformation (the result of the SMD and CMD sugar binding simulations) and the fully opened outward-facing conformation (the result of the previous long CMD and AMD equilibrium simulations) visualized the dynamics sequence for closing or opening process of the extracellular gate as depicted in Fig 4A–4C.

In the overall observed movements, for closure the bound sugar induces TM1 and TM2 from the N-terminal domain as well as TM7 from the C-terminal domain to move toward the substrate translocation pathway (Fig 4A and 4B). Specially, TM7 has two flexible kinks (XylE has only one) and the loop in the middle of the TM 7 (residue 287–295) appears to act like a lid as it reorients itself to lie directly above the sugar, closing the extracellular vestibule to occlude the sugar (Fig 4B). Mutagenesis of residues forming this loop followed by functional studies indicated that this region of GLUT1 is critical for transport. In particular, I287C, N288C, A289C, Y293I, S294A and T295A mutants showed significantly reduced transport activities [59–61]. Then TM1 and TM7 seem to move further toward the substrate translocation pathway to completely close the extracellular vestibule, changing into the inward-facing conformation (Fig 4C). A notable discrepancy between the outward-facing model and the inward-facing X-ray structure exists in the exofacial loop 1 between TM domain 1 and 2. Interestingly, this large loop comprises the single N-linked glycosylation sequence NQT (residue 45–47). The glycosylation on GLUT1 seems play an important role in sugar uptake. Deglycosylation of GLUT1 caused about 50% decrease in sugar transport and a 2.5-fold decrease in its affinity for glucose [62]. Unfortunately, the GLUT1 was crystalized in deglycosylated state by introducing a point mutation on the glycosylation site [18]. The precise structural role of glycosylation on the loop1 and its dynamics in sugar uptake of GLUT1 needs to be further investigated.

### Targeted molecular dynamics simulations for a large conformational transition in GLUT1

To investigate a large-scale structural transition and the mechanism of substrate translocation to the intracellular side, targeted molecular dynamics (TMD) simulations were employed. Each α-carbon was forced to gradually change toward the target structure, which was the X-ray structure of the human GLUT1 in the inward-facing open conformation, with a force constant. The Cα RMSD value between the starting and target structure was started from ~ 6.0 Å and ended up to ~ 1.0 Å for 10ns TMDs (residues from 9 to 455 only). To ascertain that the TMD system is reliable and reproducible, three trials with random seeds were performed, resulting in the very similar results (S5 Fig).

**Fig 5:**
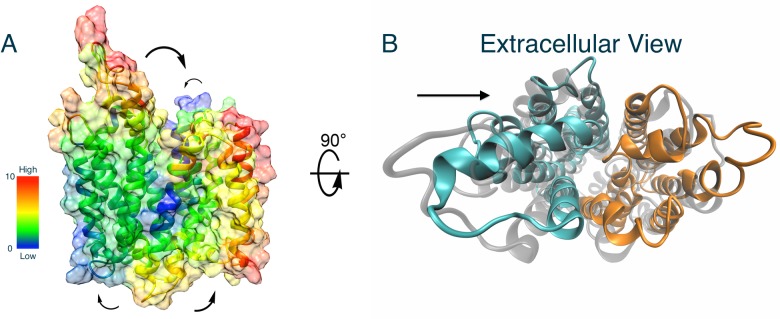
Non-symmetrical rocker-switch movement between N- and C-domain in GLUT1 during the TMD simulations. (A) Surface and cartoon presentation of the initial outward-facing conformation with color code based on the distance of each Cα atom that navigated during the TMD simulations. (B) Superimposed view of the initial partially occluded outward- (transparent gray) and the final fully opened inward-facing conformation without structural alignment. The N-domain and C-domain are shown in cyan and orange, respectively. For clarity, the intracellular domains and the C-terminal loop domain are omitted.

Our TMD analysis suggests that GLUT1 undergoes a non-symmetric rocker switch movement for substrate delivery across membranes. Overall, the extracellular N-terminal domain moves toward the C-terminal domain, which is relatively less mobile (Fig 5B).

On the intracellular side of GLUT1, both the N- and C-terminal domains shift away from one another but the C-terminal domain undergoes a larger movement than the N-terminal domain. This movement was even more clarified with monitoring atomic drifts during the TMD simulations. Each distance of equivalent α-carbon atom between the initial structure (partially occluded outward-facing conformation) and the final structure (fully opened inward-facing conformation) of the TMD were measured without structural alignment (The linker between the N- and C-terminal domains in the intracellular side and the C-terminal loop were not included in this calculation) (Fig 5A). It shows that the C-terminal domain at the interface between N- and C-terminal domains is highly stationary (mainly TM8 and a part of TM11) but the surface area that might interact with membrane is highly moveable (mainly TM9), while the interface between two domains is relatively mobile but the surface toward membrane is rather steady in the N-terminal domain.

**Fig 6:**
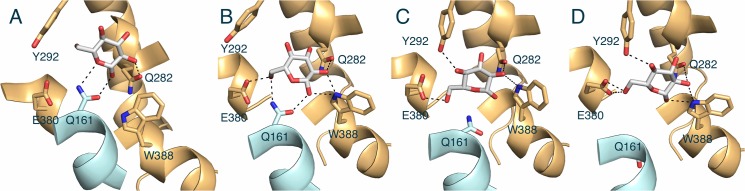
Sugar translocation pathway in GLUT1. Representative snapshots for the translocation of glucose and involved residues in TMD. Putative hydrogen bond interactions are in dash lines. The N-domain and C-domain are shown in cyan and orange, respectively.

As the outward-facing conformation transforms toward the inward-facing conformation, the TMD simulation also allowed us to visualize a translocation of D-glucose substrate from the sugar-binding site toward an inward gate, exposing the sugar to the intracellular side (Fig 6A–6D).

Generally, Gln161 underneath the glucose facilitates its movement down toward the inward gate without a change of orientation. As the hydrogen bonds between glucose and Asn288 are weakened, glucose moves down with the help of Gln161 at the N-terminal domain from the binding pocket. Then Glu380 and Tyr292 appear to form new hydrogen bonds with O6 and O4 oxygen of glucose, respectively, in concert with Gln161, Gln282, and Trp388 (Fig 6B). Gln161 moves further apart, seeming to open the sugar-releasing pathway to the intracellular side (Fig 6C and 6D). Based on the previous mutagenesis studies, Gln161 is a particularly critical residue for glucose transport [61, 63]. Mutants Q161L and Q161N showed reduced transport activity over 50- and 10-fold, respectively. Mutations of Y292 showed more modest effects but it is clear that Y292 contributes to sugar translocation. Mutants Y292F and Y292C showed about 2- and 4-fold reduced GLUT1 function, respectively [61, 63]. In a sense, Tyr292 may contribute to glucose binding with both hydrophobic and hydrophilic interactions. The close contact between the methylene group on C6 of the glucose and the phenol ring of the Tyr292 observed in our simulations could explain the hydrophobic contribution. Also the TMD data accounts for the low level of glucose uptake activities observed for mutants Q282L, W388C and E380C in previous experiments [64–66].

**Fig 7:**
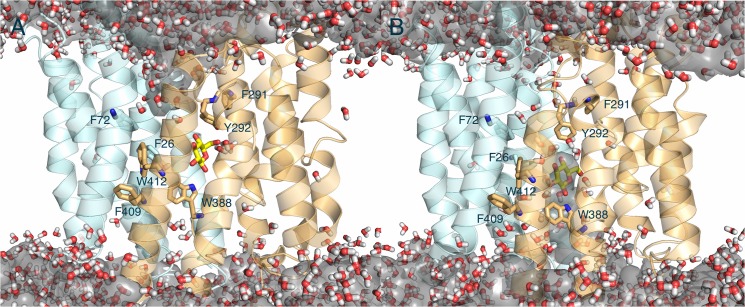
Water transport with substrate through GLUT1. Representative snapshots of (A) the outward- and (B) the inward-facing conformation through the GLUT1 tunnel during TMD simulations. Water pathways are shown in ball and stick and gray surface map. Glucose molecule is colored in yellow. The water is always isolated either from the extracellular or the intracellular side at any given time so charged molecules could not pass through the transporter during the transport cycle.

Water permeability through facilitative glucose transporters has been described previously in diffusion studies by expressing GLUT1 in *Xenopus laevis* oocytes [67]. We examined the water-filled pathway in GLUT1 in our TMD simulations. Overall, the pore-lining hydrophobic residues may involve not only in sugar translocation but also in a hydrophobic seal associated with an internal water gate in GLUT1. Two sets of hydrophobic residues appear to form each hydrophobic water barrier in the intracellular side and the extracellular side, respectively: (1) Residues Phe409, Trp388, and Trp412 below the glucose-binding site (intracellular water gate, Fig 7A); (2) Residues Phe26, Phe72, Phe291, and Tyr292 above the glucose-binding site (extracellular water gate, Fig 7B).

Particularly, F26 and Y292 in the extracellular side and W388 in the intracellular side show a large translocation during the conformational changes and may play a key role as a keeper for opening or closing the each water gate. In the final step, the glucose translocates down toward intracellular side together with many water molecules that were isolated from the extracellular side. But at any given moment a continuous water wire could not be formed because the water filled vestibules are either isolated from the extracellular or intracellular milieu by the hydrophobic residues.

### Steered molecular dynamics (SMD) simulations for sugar release

**Fig 8:**
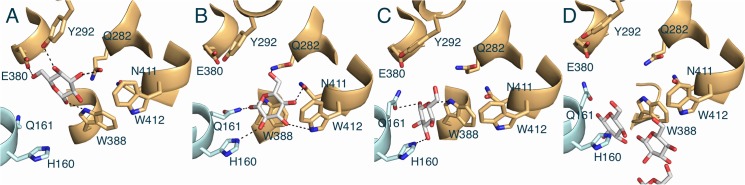
Sugar-releasing pathway in GLUT1. Representative snapshots of the glucose movement from the inward binding site along the tunnel into intracellular side in SMD. Putative hydrogen interactions are in dash lines. The N-domain and C-domain are shown in cyan and orange, respectively. The last (D) describes the further glucose path through the tunnel toward the intracellular side.

Following the transition to the opened inward-facing conformation, the glucose in the newly formed binding site at the inward gate is readily diffusible to the intracellular side. In the SMD simulations, the glucose at this stage was pulled down toward the intracellular side with an external force in a constant rate from the last snapshot of the previous TMD. During the SMD simulations, glucose substrate moves downward step-by-step with the help of Trp412, Asn411, and His160 into a new sugar-releasing pocket at the inward gate and subsequently moves down into the intracellular side. First, the hydrogen bonds between glucose and Tyr292, and Glu380 are weakened so that glucose moves downward and forms new hydrogen bonds with Trp412 and Asn411 (Fig 8A and 8B).

Next, His160 and Gln161 underneath provide new hydrogen bonds to the glucose to facilitate its movement further down into a new sugar-releasing pocket formed by His160, Gln161, and Trp388 at the inward gate (Fig 8B and 8C). Then, the glucose moves down through the relatively hydrophobic tunnel and exit into the intracellular side (Fig 8D). Previous cysteine-scanning mutagenesis studies strongly support this glucose translocation mechanism. All cysteine mutants (Q282[61], W412[57], N411[57], H160[68]) displayed significantly reduced transport activity relative to the native GLUT1.

**Fig 9:**
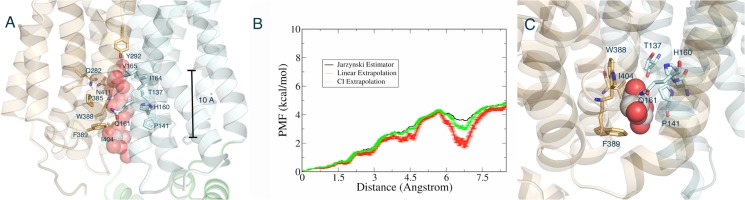
The movement of D-glucose toward the intracellular side by Steered Molecular Dynamics (SMD) simulations. (A) Representative snapshots of D-glucose movement along the tunnel. D-glucose molecules from different snapshots are shown as white and red large spheres. Contact 15 residues in GLUT1 that contributes to D-glucose binding free energy are shown. Side bar represents pulling distance of D-glucose along the *z*-axis from the sugar-release pocket corresponding to the PMF profile in (B). (B) Potential of Mean Force (PMF) profile as a function of the movement of sugar along the *z*-axis toward the intracellular side. Jarzynski estimator (black), liner extrapolation (red), and cumulative integral (CI) extrapolation (green) are shown for PMF average and standard deviation. The PMF profile computed using 10 SMD simulations from a conformational ensemble. (C) the putative transient binding site that may cause a shoulder or a local minimum at pulling distance of ~7Å on the PMF profile.

Totally ten trials were performed with random seeds, assuming that we can extract equilibrium free energy from the non-equilibrium calculations. Three different methods, Jarzynski’s equation[44], linear extrapolation[46] and cumulative integral (CI) extrapolation[46], were used and compared to construct the potential of mean force (PMF) from SMD simulations. These two extrapolation methods have been shown to provide more reliable free energy estimate than the direct Jarzynski’s equation [46]. The CI extrapolation method could obtain good estimates of free energy using about 5 to 40 fold less data set, which corrects for limited sampling of the work distribution. The PMF curve computed is displayed in Fig 9B.

At the tunnel exit point, the PMF showed a total energy barrier of ~4.5 kcal/mol. This energy barrier may correspond to the transient polar and nonpolar interactions between glucose molecule and amino acid residues located in the tunnel (S1 and S2 Tables and Fig 9A). The PMF curve also shows a shoulder or a local minimum at pulling distance of ~7Å, indicating that there might be a transient stable state along the sugar release pathway (Fig 9B). This distance is consistent with the presences of residues, W388, H160, Q161, T137, P141, I404, and F389 that showed the highest contributions on the sugar binding free energy in S1 and S2 Tables. The putative transient sugar-binding site on the sugar-releasing pathway is shown in Fig 9C. The previous cysteine-scanning mutagenesis studies showed relatively mild decrease of the transport activities with T131C[69], P141C[69], and I404C[57]. However, F389C displayed a significant loss of the transport activity[57], implying that it may play an important role in the sugar-releasing mechanism.

## Conclusions

The GLUT1 model in the outward-facing conformation presented here together with the calculated dynamics account well for previously published biochemical, mutagenesis and functional studies. They appear to be successful at not only describing the global movements of GLUT1 but also in defining key residues involved in the binding and translocation of sugar through the transporter. In addition, they agreed with the structural and functional role of ICH domain that was previously proposed. These studies are useful for our understanding of GLUT1 function and should be useful in future biochemical and drug design studies to combat devastating disease such as diabetes and cancer.

## Supporting Information

S1 Fig Structural overlay of the inward-facing open model and the x-ray structure (PDB: 4PYP) in the same conformation. (A) Side view and (B) Substrate binding site and aligned residues. Model and X-ray structure are shown in green and orange color, respectively. A bound *n*-nonyl-β-D-glucopyranoside (β-NG) molecule captured in the sugar binding site of the GLUT1 crystal is described in yellow sticks. For illustrative purposes, flexible intracellular linker domains are omitted.
(TIF)

S2 Fig CMD and AMD simulations in the absence of sugar. (1) Evolution of the RMSD values compared to the initial structure during CMD and AMD simulations. The cytoplasmic domains were excluded for this calculation. The arrow indicates a time point in the simulation when the AMD was started. The simulations were repeated with random seeds (two trials are shown in black and red lines). (2) Structure overlay of the initial (cyan) and the last (orange) snapshot at the extracellular side. The major transmembrane movements were found on TM2 and TM7 (labeled), evolving to a new fully opened outward-facing conformation.
(TIF)

S3 Fig CMD simulation with glucose bound. (A) Evolution of the RMSD values depending on the initial structure. The cytoplasmic domains were excluded for this calculation. (B) Structure overlay of the initial (cyan) and the last (orange) snapshot at the extracellular side.
(TIF)

S4 Fig The changes of radius of gyration during (A) the CMD and AMD simulation without sugar and (B) the CMD simulation with a bound sugar. Radius of gyration describes the overall spread of the conformation. Only extracellular subdomain of the structure, which is defined as all parts above the sugar binding site to the extracellular side along the z-axis, was involved for these calculations.
(TIF)

S5 Fig The RMSD profile for three TMD trials against the initial structure. They are computed using only Cα atoms of residues 9–455, which were restraints as a target.
(TIF)

S1 Table Binding free energy of GLUT1 residues for D-glucose during SMD simulations. Decomposition of binding free energy, ΔG_bind_, between D-glucose and each contact residue (both backbone and side chain) was analyzed throughout a Steered Molecular Dynamics (SMD) simulation of a sugar release. Residues are ranked according to total binding free energy, ΔG_total_, and only top ranked residues are presented. Negative values indicate that the corresponding residue favorably contributes to the sugar binding free energy. The standard deviation error for each value is shown on the next right column.
(DOCX)

S2 Table Binding free energy contributed by side chain of GLUT1 residues for D-glucose during SMD simulations. Decomposition of binding free energy, ΔG_bind_, between D-glucose and the side-chain of each contact residue was analyzed throughout a Steered Molecular Dynamics (SMD) simulation of a sugar release. Residues are ranked according to total binding free energy, ΔG_total_, and only top ranked residues are presented. The standard deviation error for each value is shown on the next right column.
(DOCX)
