# Supplementary figures and images for: Molecular Dynamics Simulations of the Human Glucose Transporter GLUT1
Source: PLoS One. 2015 Apr 28;10(4):e0125361. doi: 10.1371/journal.pone.0125361 (PMC4412407; doi:10.1371/journal.pone.0125361)

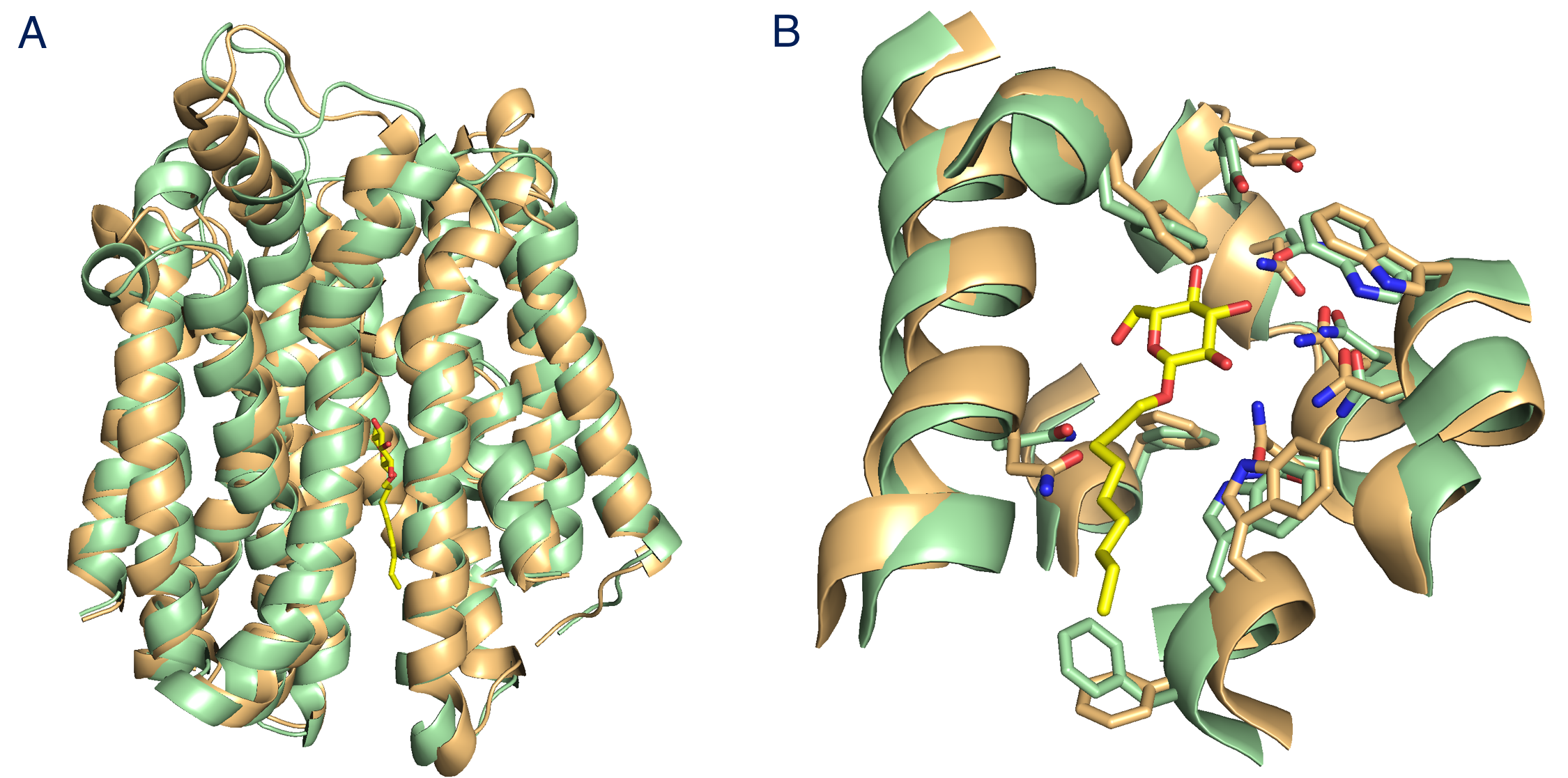

Supplement: S1 Fig — (A) Side view and (B) Substrate binding site and aligned residues. Model and X-ray structure are shown in green and orange color, respectively. A bound n-nonyl-β-D-glucopyranoside (β-NG) molecule captured in the sugar binding site of the GLUT1 crystal is described in yellow sticks. For illustrative purposes, flexible intracellular linker domains are omitted. (TIF) [file pone.0125361.s001.tif]

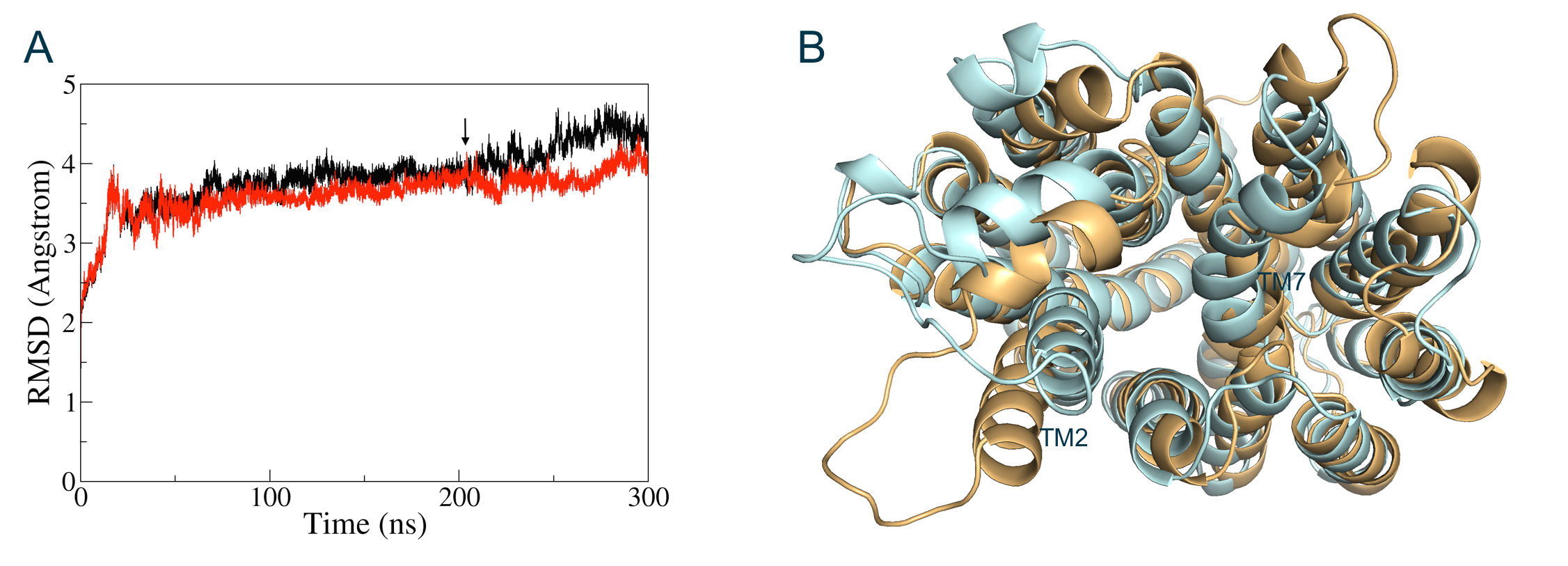

Supplement: S2 Fig — (1) Evolution of the RMSD values compared to the initial structure during CMD and AMD simulations. The cytoplasmic domains were excluded for this calculation. The arrow indicates a time point in the simulation when the AMD was started. The simulations were repeated with random seeds (two trials are shown in black and red lines). (2) Structure overlay of the initial (cyan) and the last (orange) snapshot at the extracellular side. The major transmembrane movements were found on TM2 and TM7 (labeled), evolving to a new fully opened outward-facing conformation. (TIF) [file pone.0125361.s002.tif]

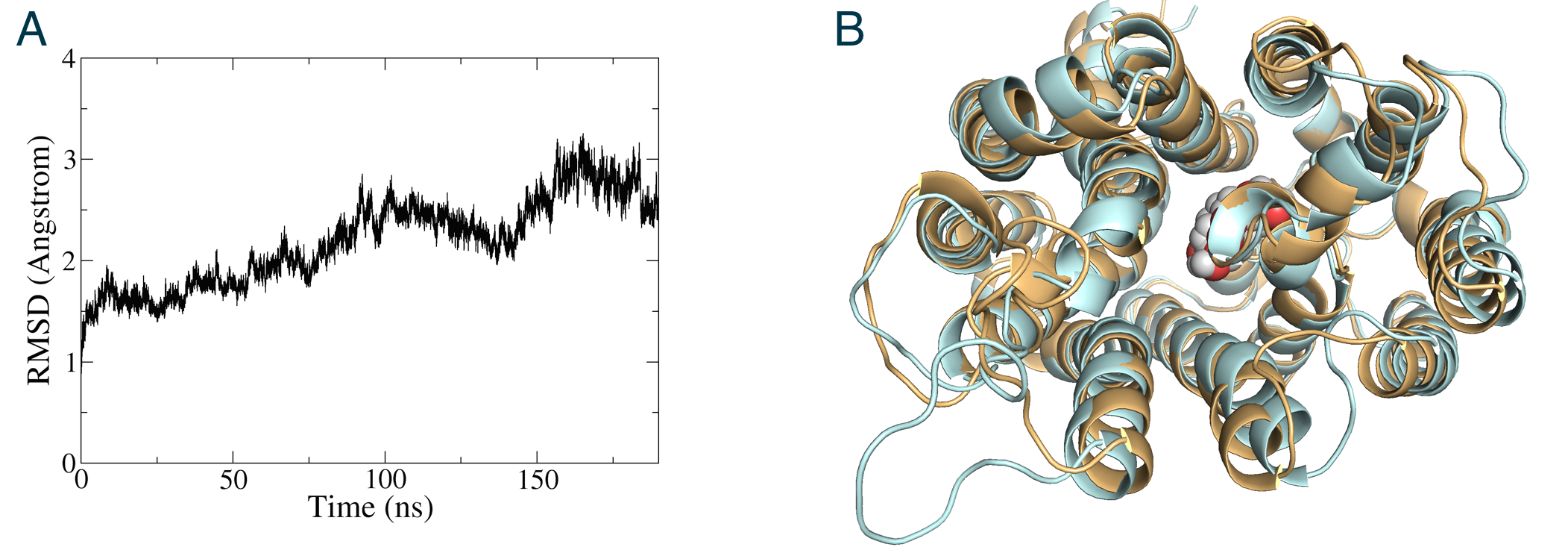

Supplement: S3 Fig — (A) Evolution of the RMSD values depending on the initial structure. The cytoplasmic domains were excluded for this calculation. (B) Structure overlay of the initial (cyan) and the last (orange) snapshot at the extracellular side. (TIF) [file pone.0125361.s003.tif]

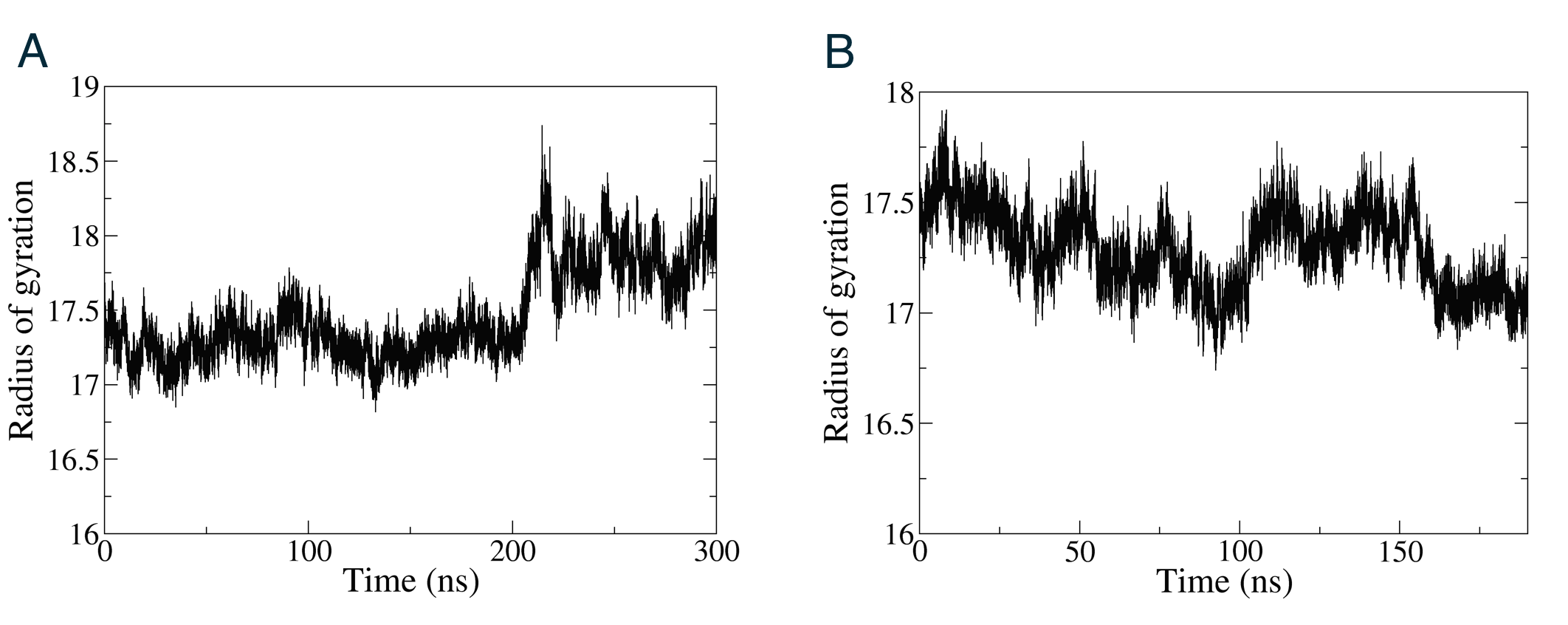

Supplement: S4 Fig — Radius of gyration describes the overall spread of the conformation. Only extracellular subdomain of the structure, which is defined as all parts above the sugar binding site to the extracellular side along the z-axis, was involved for these calculations. (TIF) [file pone.0125361.s004.tif]

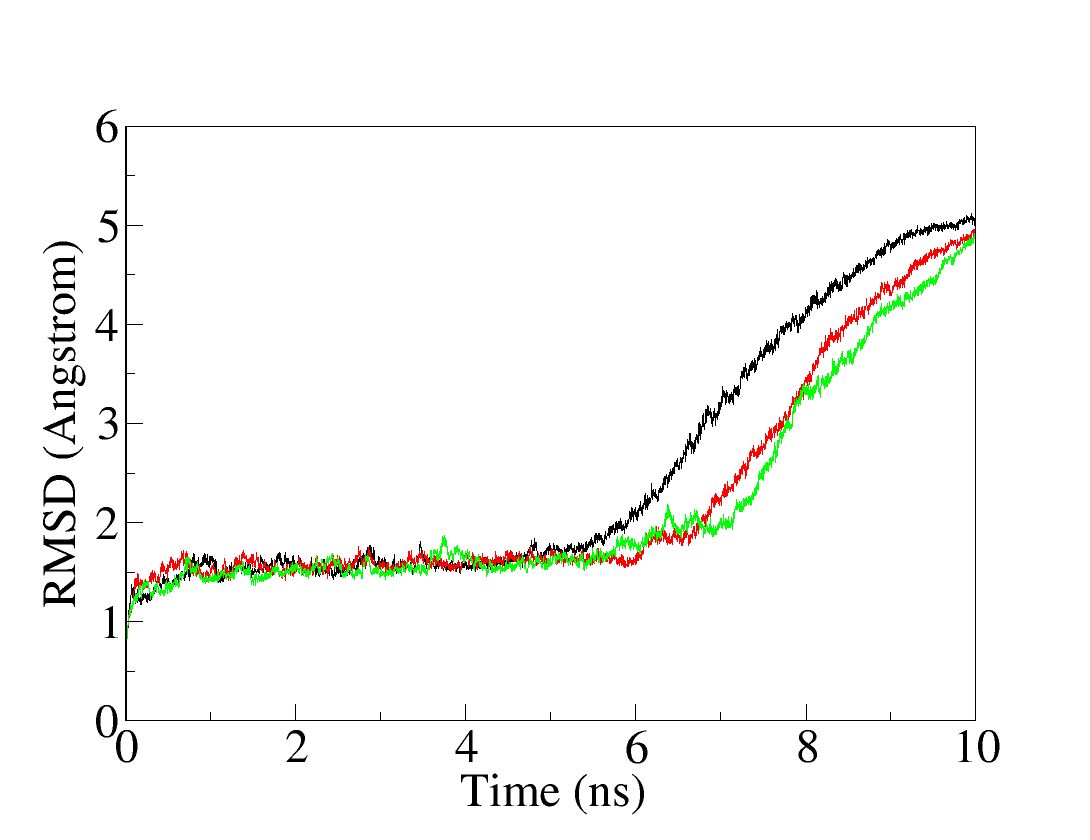

Supplement: S5 Fig — They are computed using only Cα atoms of residues 9–455, which were restraints as a target. (TIF) [file pone.0125361.s005.tif]
