# Supplementary material for: Molecular Dynamics Simulations of the Human Glucose Transporter GLUT1
Source: PLoS One. 2015 Apr 28;10(4):e0125361. doi: 10.1371/journal.pone.0125361 (PMC4412407; doi:10.1371/journal.pone.0125361)
Supplement: S1 Table — Decomposition of binding free energy, ΔGbind, between D-glucose and each contact residue (both backbone and side chain) was analyzed throughout a Steered Molecular Dynamics (SMD) simulation of a sugar release. Residues are ranked according to total binding free energy, ΔGtotal, and only top ranked residues are presented. Negative values indicate that the corresponding residue favorably contributes to the sugar binding free energy. The standard deviation error for each value is shown on the next right column. (DOCX) [file pone.0125361.s006.docx]

**S1 Table. Binding free energy of GLUT1 residues for D-glucose during SMD simulations**

| **Residue** | **Van der Waals** | **Std.Err** | **Electrostatics** | **Std.Err** | **Polar Solvation** | **Std.Err** | **Nonpolar Solvation** | **Std.Err** | **TOTAL** | **Std.Err** |
| --- | --- | --- | --- | --- | --- | --- | --- | --- | --- | --- |
| TRP 388 | -2.238 | 0.412 | -0.458 | 0.227 | 0.747 | 0.231 | -0.370 | 0.059 | -2.32 | 0.408 |
| HIS 160 | -0.824 | 0.218 | -1.180 | 0.691 | 0.803 | 0.536 | -0.155 | 0.035 | -1.36 | 0.301 |
| GLN 161 | -0.851 | 0.199 | -0.830 | 0.613 | 0.670 | 0.319 | -0.148 | 0.038 | -1.16 | 0.293 |
| ILE 164 | -0.596 | 0.205 | -0.395 | 0.398 | 0.288 | 0.281 | -0.101 | 0.027 | -0.80 | 0.160 |
| THR 137 | -0.591 | 0.116 | -0.253 | 0.250 | 0.252 | 0.195 | -0.114 | 0.027 | -0.71 | 0.137 |
| PRO 141 | -0.466 | 0.188 | -0.087 | 0.190 | 0.122 | 0.235 | -0.080 | 0.042 | -0.51 | 0.146 |
| ILE 404 | -0.336 | 0.159 | -0.094 | 0.123 | 0.096 | 0.109 | -0.074 | 0.028 | -0.41 | 0.127 |
| GLN 282 | -0.298 | 0.112 | -0.132 | 0.100 | 0.134 | 0.118 | -0.052 | 0.027 | -0.35 | 0.140 |
| PHE 389 | -0.199 | 0.106 | -0.043 | 0.148 | 0.002 | 0.088 | -0.032 | 0.018 | -0.27 | 0.094 |
| ASN 411 | -0.086 | 0.078 | -0.043 | 0.064 | -0.082 | 0.142 | -0.010 | 0.019 | -0.22 | 0.057 |
| TYR 292 | -0.145 | 0.101 | -0.017 | 0.029 | -0.015 | 0.108 | -0.016 | 0.021 | -0.19 | 0.053 |
| VAL 165 | -0.121 | 0.075 | -0.050 | 0.072 | 0.009 | 0.078 | -0.017 | 0.019 | -0.18 | 0.052 |
| PRO 385 | -0.145 | 0.100 | -0.005 | 0.041 | 0.018 | 0.104 | -0.019 | 0.022 | -0.15 | 0.049 |
| THR 158 | -0.106 | 0.061 | -0.057 | 0.098 | 0.039 | 0.089 | -0.008 | 0.008 | -0.13 | 0.045 |
| GLN 283 | -0.105 | 0.082 | 0.007 | 0.051 | -0.008 | 0.106 | -0.012 | 0.017 | -0.12 | 0.034 |
| GLN 279 | -0.081 | 0.055 | -0.006 | 0.060 | -0.011 | 0.077 | -0.010 | 0.012 | -0.11 | 0.035 |
| VAL 391 | -0.104 | 0.057 | -0.008 | 0.061 | 0.028 | 0.082 | -0.012 | 0.014 | -0.10 | 0.028 |
| VAL 140 | -0.067 | 0.040 | -0.004 | 0.036 | -0.012 | 0.030 | -0.004 | 0.006 | -0.09 | 0.031 |

Decomposition of binding free energy, ΔG_bind_, between D-glucose and each contact residue (both backbone and side chain) was analyzed throughout a Steered Molecular Dynamics (SMD) simulation of a sugar release. Residues are ranked according to total binding free energy, ΔG_total_, and only top ranked residues are presented. Negative values indicate that the corresponding residue favorably contributes to the sugar binding free energy. The standard deviation error for each value is shown on the next right column.
