# Supplementary material for: Molecular Dynamics Simulations of the Human Glucose Transporter GLUT1
Source: PLoS One. 2015 Apr 28;10(4):e0125361. doi: 10.1371/journal.pone.0125361 (PMC4412407; doi:10.1371/journal.pone.0125361)
Supplement: S2 Table — Decomposition of binding free energy, ΔGbind, between D-glucose and the side-chain of each contact residue was analyzed throughout a Steered Molecular Dynamics (SMD) simulation of a sugar release. Residues are ranked according to total binding free energy, ΔGtotal, and only top ranked residues are presented. The standard deviation error for each value is shown on the next right column. (DOCX) [file pone.0125361.s007.docx]

**S2 Table. Binding free energy contributed by side chain of GLUT1 residues for D-glucose during SMD simulations**

| **Residue** | **Van der Waals** | **Std.Err** | **Electrostatics** | **Std.Err** | **Polar Solvation** | **Std.Err** | **Nonpolar Solvation** | **Std.Err** | **TOTAL** | **Std.Err** |
| --- | --- | --- | --- | --- | --- | --- | --- | --- | --- | --- |
| TRP 388 | -1.798 | 0.777 | -0.404 | 0.207 | 0.627 | 0.309 | -0.315 | 0.129 | -1.890 | 0.802 |
| HIS 160 | -0.545 | 0.194 | -1.096 | 0.841 | 0.774 | 0.610 | -0.134 | 0.049 | -1.000 | 0.417 |
| GLN 161 | -0.585 | 0.272 | -0.558 | 0.490 | 0.447 | 0.388 | -0.130 | 0.066 | -0.825 | 0.382 |
| ILE 164 | -0.484 | 0.244 | -0.193 | 0.315 | 0.153 | 0.258 | -0.092 | 0.043 | -0.615 | 0.273 |
| THR 137 | -0.345 | 0.156 | -0.193 | 0.245 | 0.137 | 0.192 | -0.079 | 0.038 | -0.480 | 0.204 |
| PRO 141 | -0.319 | 0.130 | -0.088 | 0.091 | 0.102 | 0.110 | -0.071 | 0.033 | -0.378 | 0.153 |
| ILE 404 | -0.189 | 0.088 | -0.081 | 0.113 | 0.051 | 0.079 | -0.048 | 0.028 | -0.267 | 0.127 |
| LEU 284 | -0.223 | 0.081 | -0.025 | 0.103 | 0.060 | 0.115 | -0.036 | 0.021 | -0.224 | 0.110 |
| GLN 282 | -0.155 | 0.082 | -0.067 | 0.123 | 0.088 | 0.120 | -0.032 | 0.019 | -0.166 | 0.064 |
| PRO 385 | -0.111 | 0.045 | -0.107 | 0.081 | 0.097 | 0.069 | -0.024 | 0.014 | -0.145 | 0.031 |
| ALA 407 | -0.079 | 0.056 | -0.111 | 0.031 | 0.063 | 0.056 | -0.014 | 0.015 | -0.141 | 0.040 |
| PHE 389 | -0.134 | 0.045 | -0.022 | 0.066 | 0.055 | 0.079 | -0.025 | 0.010 | -0.126 | 0.030 |
| ASN 411 | -0.107 | 0.156 | -0.015 | 0.268 | 0.024 | 0.249 | -0.018 | 0.036 | -0.116 | 0.217 |
| GLY 163 | -0.092 | 0.035 | -0.034 | 0.063 | 0.055 | 0.063 | -0.013 | 0.009 | -0.084 | 0.014 |
| TYR 292 | -0.087 | 0.018 | -0.026 | 0.032 | 0.047 | 0.035 | -0.014 | 0.003 | -0.081 | 0.015 |
| GLY 398 | -0.068 | 0.032 | -0.016 | 0.053 | 0.023 | 0.052 | -0.018 | 0.007 | -0.080 | 0.018 |
| VAL 165 | -0.064 | 0.020 | -0.005 | 0.030 | 0.001 | 0.028 | -0.008 | 0.004 | -0.076 | 0.023 |
| THR 158 | -0.054 | 0.013 | -0.030 | 0.052 | 0.025 | 0.047 | -0.006 | 0.002 | -0.064 | 0.016 |

Decomposition of binding free energy, ΔG_bind_, between D-glucose and the side-chain of each contact residue was analyzed throughout a Steered Molecular Dynamics (SMD) simulation of a sugar release. Residues are ranked according to total binding free energy, ΔG_total_, and only top ranked residues are presented. The standard deviation error for each value is shown on the next right column.
